# Tartrazine-Induced Toxicity Is Attenuated by Naringenin in Wistar Rats: A Biochemical, Histological, and Behavioral Study

**DOI:** 10.3390/nu18193161

**Published:** 2026-09-25

**Authors:** Eduardo Rivadeneyra-Domínguez, Juan Francisco Rodríguez-Landa, David J. Salazar-Camacho, Isaac Zamora-Bello, Óscar Rosales-Sánchez, María de la Soledad Lagunes-Castro, Socorro Herrera-Meza, Jorge E. Morales-Mávil

**Affiliations:** 1 Facultad de Química Farmacéutica Biológica, Universidad Veracruzana, Xalapa 91000, Veracruz, Mexico; juarodriguez@uv.mx (J.F.R.-L.); vchido2000@gmail.com (D.J.S.-C.); iszamora@uv.mx (I.Z.-B.); osrosales@uv.mx (Ó.R.-S.); 2 Laboratorio de Neurofarmacología, Instituto de Neuroetología, Universidad Veracruzana, Xalapa 911900, Veracruz, Mexico; 3 LADISER Inmunología y Biología Molecular, Facultad de Ciencias Químicas, Universidad Veracruzana, Orizaba 94340, Veracruz, Mexico; marlagunes@uv.mx; 4 Instituto de Investigaciones Psicológicas, Universidad Veracruzana, Xalapa 91190, Veracruz, Mexico; soherrera@uv.mx; 5 Laboratorio de Biología de la Conducta, Instituto de Neuroetología, Universidad Veracruzana, Xalapa 91190, Veracruz, Mexico; jormorales@uv.mx

**Keywords:** tartrazine, food additive, naringenin, liver function tests, kidney function test, histological analysis

## Abstract

**Background**: Tartrazine (TZ) has been linked to hyperactivity in children, as well as to kidney and liver damage. Furthermore, naringenin (NG) is a flavonoid with antioxidant and anti-inflammatory properties that may mitigate the damage caused by TZ. **Methods**: Four independent groups were used, each consisting of seven male Wistar rats—vehicle VEH (saline solution/DMSO, 0.600 mL; IP), TZ (150 mg/kg; IP), NG (100 mg/kg; IP), and TZ + NG (150 mg/kg; 100 mg/kg; IP). For 28 days, treatments were administered every 24 h, and the behavioral effects were evaluated via the open field and Rotarod tests. At the end of the behavioral tests, biochemical and histological analyses of the liver, kidney, and intestine were performed. **Results**: In the open field test, TZ decreased the number of crossings and rearing behaviors, which was attenuated in NG. The number of grooming behaviors was significantly reduced by TZ, while the duration of this behavior did not significantly differ between the treatment groups. In Rotarod, the TZ group presented less coordination than the other groups. In the TZ group, all parameters included in the liver and kidney function tests showed a significant increase compared to normal levels, reflecting a pathological condition in those organs. On the other hand, groups TZ + NG were within the reference intervals. Treatment with NG reduced the damage caused by TZ. Finally, in the histological analysis, damage to liver, kidney, and intestinal tissues was observed in the TZ groups, whereas treatment with NG mitigated the cellular damage caused by TZ. **Conclusions**: Naringenin exhibited a protective effect as it restored liver and kidney parameters to normal levels and demonstrated a predominant preservation of the histological architecture of the liver, kidney, and intestine. In this regard, the protective effect observed could be related to the antioxidant, anti-inflammatory, and anti-apoptotic properties described for naringenin. However, further research is still needed to validate the use of naringenin as a potential candidate for improving renal and hepatic function, based on mechanistic studies.

## 1. Introduction

According to the World Health Organization (WHO), since 2016, the acceptable daily intake (ADI) of TZ has been set at 0–10 mg/kg of body weight [1]. Despite being one of the first food additives linked to potential adverse reactions, TZ remains authorized for use in the European Union (EU) and other regions [2]. Tartrazine, classified as E102 according to the European coding system for food additives, is a dye used in a wide range of processed foods and even in the pharmaceutical industry [3]. Although direct evidence linking TZ to anxiety in humans is limited, preclinical studies suggest that TZ increases oxidative stress (OS), negatively affects liver and kidney function, alters lipid levels, and, as a result, increases the risk of cardiovascular disease [4,5]. Data on neurobehavioral effects are often inconsistent. Findings regarding its impact on behavior and neurotoxicity are often contradictory or inconclusive. Most preclinical studies demonstrating the toxic effects of TZ have used doses ranging from 100 to 700 mg/kg body weight in rodents [6,7,8,9,10,11,12,13], which is considerably higher than typical dietary exposure in humans (0–10 mg/kg body weight) [1]. While these high doses are useful for elucidating the mechanisms of toxicity, their direct relevance to humans is limited. Nevertheless, they remain important for risk assessment, as cumulative exposure from multiple sources (food, pharmaceuticals, cosmetics) may approach or exceed the acceptable daily intake (ADI), especially in children [14]. Furthermore, a study conducted in Kuwait in 2008 showed that tartrazine consumption substantially exceeded the acceptable daily intake, especially among young children, a population group considered highly vulnerable to the harmful effects of food dyes [15]. Another study highlighting the link between tartrazine consumption and health disorders is that presented by Sasaki et al. (2002) [16], who found that tartrazine induced DNA damage in the colons of mice; furthermore, this food coloring may also cause DNA damage in the liver and kidneys, according to Hassan (2010) and Khayyat et al. (2017) [17,18]. Currently, measures to reduce consumption of and exposure to tartrazine focus on four areas: labeling warnings, establishing acceptable intake limits, monitoring consumption among vulnerable populations, and reformulating products using alternatives; national health authorities and organizations such as EFSA and JECFA are involved in this process [4]. Recent evidence also emphasizes that monitoring of vulnerable populations must be strengthened, especially among children, as concerns regarding exposure and accumulation through diet are concentrated in this group [19].

The toxicity of TZ may be due to its direct covalent binding to proteins, which causes denaturation of the proteins’ active sites and an abnormal conformation [20]. Metabolic biotransformation of the azo bond can generate reactive amines, which, in turn, generate free radicals and exacerbate oxidative damage—one of the main factors determining TZ toxicity [21,22]. In a study conducted by Wopara et al. (2021), the researchers demonstrated that rats treated with TZ exhibited higher levels of TNF-α, IL-1β, and IL-6 in the liver [23]. In the same study, a significant increase in the expression of the testicular genes for TNF-α, IL-1α, and IL-1β was reported in rats which had been administered TZ and erythrosine. Furthermore, TNF-α is an essential cytokine that regulates various processes in the mammalian liver. It can also promote the release of IL-1β and IL-6, which can induce oxidative stress [24]; thus, oxidative stress and inflammation are interrelated mechanisms. Accordingly, it has been shown that excessive ROS production activates nuclear factor kappa B (NF-κB), a transcription factor that can mediate the upregulation of genes encoding the synthesis of proinflammatory mediators and is considered the primary regulator of inflammation [25]. Therefore, TZ-induced liver inflammation may be mediated by ROS-induced upregulation of NF-κB and the resulting increase in the synthesis of gene products, such as inflammatory cytokines. Raposa et al. (2016) noted that TZ could significantly increase NF-κB expression in the livers of male and female mice [26]. Furthermore, lipid peroxidation can reduce membrane fluidity and impair membrane integrity and function, leading to significant pathological changes [27]. From a biological and mechanistic perspective, oxidative stress (OS) has been identified as a key pathway mediating TZ toxicity, contributing to an imbalance between pro-oxidants and antioxidants in cells [28,29]. This imbalance is reflected in elevated levels of malondialdehyde (MDA), a marker of lipid peroxidation, indicating increased oxidative damage. At the same time, endogenous antioxidant enzymes, such as superoxide dismutase (SOD) and catalase (CAT), are often significantly reduced, supporting the shift toward a pro-oxidant state induced by TZ exposure [30].

On the other hand, naringenin is a citrus-derived flavanone with attributed antioxidant, anti-inflammatory, and anti-apoptotic properties, and has been evaluated in various experimental models of liver damage induced by chemical agents and drugs. Zaidun et al. (2018) noted that this flavanone participates in the modulation of processes associated with oxidative stress and cellular damage [31]. In preclinical models, its hepatoprotective effect has been linked to a decrease in hepatocellular injury enzymes, modulation of oxidative markers, reduction of inflammatory mediators, and preservation of liver architecture. For example, Wang et al. (2014) evaluated naringenin in a murine model of acute liver injury induced by carbon tetrachloride and reported a decrease in ALT and AST, as well as an improvement in hepatic histological alterations [32]. Therefore, the objective of this study was to evaluate the protective effect of naringenin against tartrazine-induced toxicity in Wistar rats. These effects were evaluated through behavioral tests, liver and kidney function tests, and histological analysis. The results obtained in this study may be useful for conducting a future evaluation of a potential herbal medicine or pharmaceutical formulation based on this flavanone, aimed at reducing liver and kidney damage associated with the administration of drugs or food additives with potential hepatotoxic and/or nephrotoxic effects.

## 2. Materials and Methods

### 2.1. Subjects and Experimental Design

Twenty-eight Wistar rats weighing between 250 and 300 g at the start of the experiment and aged two months were included. The animals were divided into 4 groups of 7 individuals and housed in translucent acrylic boxes (44 × 33 × 25 cm) in the vivarium of the Pharmacotoxicology Laboratory of the Faculty of Biological Pharmaceutical Chemistry, Xalapa region, at a temperature between 26 ± 2 °C and a light/dark cycle of 12 × 12 h. These animals were given access to purified water and food ad libitum. The experimental subjects were randomly assigned to different groups using an online free program (https://random.org). The sample size per group (n = 7) was based on previous studies that reported that 6 to 8 rats per group were sufficient to detect behavioral effects in the test used in the present study without compromising the statistical power [33,34]. This was also considering the recommendations of the ethics committee to apply 3R principles of pre-clinical research [35]. Regarding blinding, only the project director was aware of the treatment assignments, and the rest of the researchers and staff who conducted the behavioral and biochemical tests and the histological analysis were blinded to the treatment.

The experimental subjects were distributed as follows: VEH (saline solution/DMSO, i.p.), TZ (150 mg/kg, i.p.), NG (100 mg/kg, i.p.), and TZ + NG (150 mg/kg and 100 mg/kg, i.p.). The maximum volume administered to each rat per dose was 2 mL. All treatments were administered every 24 h for 28 days, without interruption. On the 28th day of treatment, between 8 and 9 a.m., the Rota-rod and open-field behavioral tests were conducted. Furthermore, none of the study subjects exhibited any health problems, a fact that was verified daily using an ethogram; consequently, none of the study subjects died, and the experiment was thus successfully completed. Upon completion of the behavioral tests, the rats were placed under deep anesthesia with sodium pentobarbital (120 mg/kg, i.p., Cheminova de México, Mexico City, Mexico; SAGARPA Reg. No. Q-0449-124). The absence of eyelid reflexes and pinch reflexes in the paws or tail was confirmed, as was the absence of pain or distress and the presence of unconsciousness. Blood samples were obtained via cardiac puncture using a 3 mL syringe fitted with a 22 × 32 mm needle. The blood was then transferred to a tube without anticoagulant and, once clotted, centrifuged at 3500 rpm for 5 min.

On the other hand, it is important to mention that in preclinical research, and particularly in murine models, this route of administration is used to evaluate pharmacokinetics, metabolism, and—for this study in particular—toxicity. Furthermore, when administered intraperitoneally, approximately 90% of the tartrazine is absorbed directly into the bloodstream, whereas oral absorption is less than 5%. As for naringenin, it has low oral bioavailability, which is why the intraperitoneal route was selected.

### 2.2. Dose

The dose of naringenin (Sigma Aldrich^®^, St. Louis, MO, USA; CAS 67604-48-2) was 100 mg/kg per day intraperitoneally for 28 consecutive days, as it has been shown to have a hepatoprotective effect according to studies by Arafah et al. (2020) [36]. Naringenin has demonstrated hepatoprotective and nephroprotective activity in preclinical models using Wistar rats exposed to acetaminophen, gentamicin, and cisplatin [8,37,38,39,40,41,42,43]. However, most of these studies involved acute or subacute regimens. Since the present study required a 28-day subchronic regimen with concomitant administration of tartrazine, a dose of 100 mg/kg administered intraperitoneally was selected, with the aim of maintaining sustained exposure to the flavonoid throughout the experimental protocol.

The dose of tartrazine (tartrazine, T0388-100G; dye content ≥ 85%; acid yellow 23; Sigma Aldrich^®^, St. Louis, MO, USA; Code: 1003266441) was 150 mg/kg per day intraperitoneally for 28 consecutive days, in accordance with studies conducted by Matayoshi (2017) [44]; Ahmad et al. (2019) [45]; and Balta (2019) [46] indicated that toxicity and liver damage occur at this dose. Another important reason for using both doses was to avoid creating a dose–response curve, thereby aligning with the “3Rs” principle for animal research and minimizing the number of experimental subjects, as well as adhering to these ethical principles. Regarding the vehicle solution, it consisted of saline and DMSO (dimethyl sulfoxide p.a. EMSURE^®^ ACS, 9:1, Reference: 1.02952, Item Number: 102952, 3050 Spruce St., St. Louis, MO 63103, USA; Brand: Millipore, CAS No.: 67-68-5). DMSO was chosen as part of the vehicle because it is a good solvent for tartrazine. The compounds (naringenin and tartrazine) were stored in accordance with the manufacturer’s recommendations and specifications (at room temperature of 22 °C, in a dry place protected from moisture, without exposure to light) until use: it is important to emphasize that the solutions were administered to the experimental subjects immediately after preparation. The solutions were prepared in 15 mL amber glass vials protected from direct UV light. To prepare this solution, the total number of experimental rats—in this case, 28—was taken into account. For each rat, 2 mL of vehicle was used to create a daily stock solution. Using a 200–1000 µL micropipette with a separate tip for each reagent, the solution was transferred to an amber bottle (as DMSO is photosensitive). For the tartrazine dose, 300 mg of tartrazine was diluted in 2 mL of 0.09 per cent sterile saline solution from PISA and homogenized in a Cole-Parmer vortex (Toronto, Canada; Item no. 04726-01) mixer for 2 min to obtain a concentration of 150 mg/mL (referred to as the ‘stock solution’), which was subsequently administered to the experimental subjects, taking body weight into account using a rule of three (for example, a rat weighing 300 g was administered 0.3 mL of the working solution intraperitoneally). In the case of naringenin, 200 mg of the compound was diluted in 1.8 mL of 0.09 per cent sterile saline solution (PISA Farmacéutica^®^, Ciudad de México, México), plus 0.2 mL of DMSO, and homogenized in a Cole-Parmer vortex mixer for 2 min, until a concentration of 100 mg/mL was obtained (referred to as the ‘stock solution’); this was then administered, taking into account the body weight of the experimental subjects using a rule of three (for example, a rat weighing 300 g was administered 0.3 mL of the working solution intraperitoneally).

### 2.3. Behavioral Tests

Sixty minutes before starting the behavioral tests, the subjects were carried out and maintained in the experimental room to acclimatize them to the novel surroundings. The behavioral test was performed between 10:00 and 13:00 h. Each rat was evaluated once in the locomotor activity test (5 min), and subsequently in the Rotarod previous training. A digital video camera (Sony DCR-SR42, 40x optical zoom, Carl Zeiss lens, San Diego, CA, USA) was installed above the locomotor activity cage to record the behavioral activity. The activity on the Rotarod was automatically recorded. At the end of behavioral test, two blind independent observers quantified the behavioral variables from video-recorded sessions until a 95% agreement was obtained, which was performed with an ex professo software version 1.0.

#### 2.3.1. Open Field Test

Each rat was placed in a translucent plexiglass cage (20 cm height, 33 cm × 44 cm base) for 5 min. The floor was divided into 12 squares of 11 cm × 11 cm each, where the variables (a) number of crossings, (b) number of rearing behaviors, (c) time of rearing behavior, and (d) time of grooming behaviors was evaluated according to Rivadeneyra-Domínguez et al. (2023) [47]. Subsequently, the Rotarod test was performed.

#### 2.3.2. Rotarod Test

The Rotarod’s rotating rod was set to 18 rpm; therefore, to conduct this test, all rats were trained on the apparatus for 6 consecutive days for 5 min. Before placing the rats on the Rotarod for the test, the cylinder was set in motion; the rat was placed on the roller by holding it by the tail; and the rats’ motor activity was then assessed by measuring the latency period during which the rat remained walking on the roller, until it fell off; the timer was stopped, and the observed results were recorded [48]. This test was performed to identify changes in balance and motor coordination. The parameter measured was the fall latency and was recorded automatically via a Rotarod LE 8300 (PanLab Scientific Instruments, Letica LSI, Barcelona, Spain).

### 2.4. Biochemical Tests

The parameters used for liver function tests were gamma glutamyl transferase (GGT), total protein (TP), alkaline phosphatase (ALP), aspartate aminotransferase (AST), alanine aminotransferase (ALT), total bilirubin, direct bilirubin, and indirect bilirubin. Renal and liver function tests were performed using commercial kits. The commercial kits were manufactured by SPINREACT^®^ Lab, Center de México, S.A. de C.V. Once the blood samples were collected, they were transferred to dry (anticoagulant-free) Vacutainer tubes, allowing the blood to clot, then centrifuged at 3500 revolutions per minute for 5 min to obtain serum, which was immediately transferred using a Pasteur pipette to the appropriate containers for the specified dry chemistry tests on the KONTROLab Italia Ltd. instrument. Thus, the samples were processed on the same day on which they were collected.

The kits used were based on both colorimetric and kinetic methods; the former relies on a color change at the end of a reaction (end point), while the latter evaluates the rate of optical change over time (absorbance per minute), which is ideal for measuring enzymatic activity. The sensitivity of a colorimetric method refers to its ability to detect small changes in the concentration of a substance through color variation, whereas the sensitivity of a kinetic method is its ability to detect small variations in the concentration of a substance by measuring the rate at which a chemical or enzymatic reaction occurs.

The results obtained were compared to the biological reference ranges for Wistar rats; these ranges were established based on the strain, sex, and conditions of our laboratory, which is why these ranges were used in other studies where this type of testing had been conducted.

### 2.5. Histological Analysis

The organs (liver, kidney, and intestine) of Wistar rats were removed as a “cluster” and fixed at room temperature (22–23 °C) with 4% paraformaldehyde at pH 7.4. Forty-eight hours later, the organs were separated, and biopsies measuring 1 × 1 cm were collected from the kidneys and liver and 0.5 cm from the intestines. The abdominal organ biopsies were washed with 0.1 M PBS (pH 7.4) and post-fixed with 10% formalin for 72 h for subsequent paraffin embedding using an automated system (Histokinet Inc. ©, Ramsey, Minnesota 55303, USA). From paraffin blocks, serial sections 5 µm thick were prepared; for the organs, sections were made longitudinally for the liver and kidney and transversely for the intestine. The sections were deparaffinized with xylene, followed by heating for 30 s in a histology oven at 60 °C. Prior to hematoxylin–eosin staining, the sections were rehydrated using a gradient of 100% ethanol (twice), 96% ethanol (twice), 80% ethanol (once), and distilled water. After this process, the sections were stained with Gill’s hematoxylin (Cat. 234-16; Sigma-Aldrich, St. Louis, MO, USA) for 3 min; they were washed with double-distilled water, and then the color was changed from purple to blue using a 0.05% lithium carbonate solution for 10 s. They were rinsed with water, and then eosin was added for 1 min. At the end of this process, the tissues were dehydrated using an ethanol gradient—70%, 80%, 96% (twice), 100% (twice)—followed by ethanol–xylene (50:50) and xylene (three times). The slides were coated with histological resin (Entellan, Sigma-Aldrich, St. Louis, MO, USA) and finally covered with coverslips. The images were captured using a Leica DM1000 microscope (Wetzlar, Germany) and digitized with a Leica DC450 camera (Wetzlar, Germany). Image analysis focused on digital color removal using a fractal-type algorithm with ImageJ software (version v1.54t), followed by a grayscale image segmentation protocol using Adobe Photoshop. The scoring scale was as follows: necrotic foci, signs of apoptosis, cellular infiltrate, hypochromic nuclei, hyperchromic nuclei, hypochromic cytosol, hyperchromic cytosol, cellular hypotrophy, cellular hypertrophy, presence of erythrocytes, microvascular congestion, and microvascular atrophy.

Histological analysis was performed on the 28 experimental individuals. Five histological sections were obtained per individual, of which 20 fields per histological section were analyzed using a 100x objective of an optical microscope, resulting in a total of 560 fields.

### 2.6. Statistical Analysis

The assumption of the normality of the data were verified with Shapiro–Wilk test and then the data were analyzed as follow: data that satisfy the assumption of normality and homogeneity were analyzed by one-way ANOVA for independent groups, with treatment as the only factor, and Student-Newman-Keuls as the post hoc test. Contrarily, one-way analysis of variance on ranks was performed, and as post hoc test Dunn. The level of significance was set at *p* < 0.05. The results are presented as the mean ± SEM. All analyses were conducted using GraphPad Prism (version 8c; San Diego, CA, USA).

## 3. Results

### 3.1. Behavioral Tests

#### 3.1.1. Open Field Test

##### Number of Crossings

Analysis of the number of crossings revealed significant differences between the treatment groups [F_(3,24)_ = 7.294; *p* = 0.001]. Post hoc tests showed that the TZ-treated group had fewer number of crossings than the VEH group. This effect was attenuated by treatment with NG (TZ + NG group). NG per se did not produce significant change on this variable (Table 1).

##### Number of Rearing Behaviors

The analysis of the number of rearing behaviors, revealed significant differences between the groups [F_(3,24)_ = 8.048; *p* = 0.001]. Post hoc tests revealed that this variable was lower in the group treated with TZ in comparison with the VEH group. Similarly, the NG and TZ + NG significantly reduced this variable in comparison with the VEH group.

##### Time Spent Rearing Behavior

No significant differences were found in the time spent rearing between the treatment groups [F_(3,24)_ = 1.763; *p* = 0.181]. However, post hoc test revealed that the TZ group had the higher time spent in this behavior, compared with the VEH, NG, and TZ + NG groups (Table 1).

##### Number of Grooming Behavior

The number of grooming behaviors showed significant differences between the treatment groups [H(3) = 14.723, *p* = 0.002]. Post hoc tests revealed that this variable was lower in the group treated with TZ than in the VEH, NG, and TZ + NG groups. However, this effect was not prevented by the combined treatment with NG (Table 1).

##### Time Spent Grooming Behavior

The analysis of this variable showed no significant differences between treatment groups [H(3) = 1.497, *p* = 0.683], see Table 1.

#### 3.1.2. Rotarod

##### Latency to Fall

The statistical analysis revealed significant differences between the treatment groups [F_(3,24)_ = 6.108; *p* = 0.003] in the latency to fall. Post hoc tests revealed that this variable was significantly lower in the group treated with TZ than in the VEH group. This effect of TZ was attenuated by NG (Table 1).

### 3.2. Biochemical Tests

Liver and kidney function tests revealed significantly greater values in the groups receiving TZ than in the other groups. The elevated liver enzymes are as follows: gamma-glutamyl transferase (GGT), total protein (TP), alkaline phosphatase (ALP), AST (aspartate aminotransferase), ALT (alanine aminotransferase), total bilirubin, direct bilirubin, and indirect bilirubin. The elevated renal parameters are as follows: urea and creatinine. (Table 2 and Table 3). In fact, the results for the parameters that make up liver and kidney function in the NG group fall within normal levels or reference ranges.

### 3.3. Histological Analysis

This first section presents the analysis of the liver histological sections (Figure 1), in which the VEH group revealed a normal architecture of the central hepatic vein, which is located at the center of each lobule and surrounded by strands of hepatocytes. Regarding these and the sinusoidal cells, they retain both their cellular integrity and their structure (Figure 1a). As for the TZ group (Figure 1b), dilation of the central hepatic vein was observed, along with the presence of infiltrating cells and microvascular atrophy, as well as a disorganization of the hepatocyte cords, revealing alterations including, notably, signs of apoptosis, hypochromic nuclei, and cellular hypotrophy. The findings described above suggest an increase in transaminases (AST, ALT), GGT, and ALP (Table 2). In the NG group (Figure 1c), there were neither structural changes in the central hepatic vein nor any cellular alterations in the hepatocytes. Microphotographs of liver sections from rats treated with TZ + NG revealed that the hepatocytes appeared virtually normal, and that there was slight dilatation of the central hepatic vein, as well as the presence of erythrocytes (Figure 1d).

The second section presents an analysis of the renal histological sections (Figure 2), in which the VEH group revealed a normal distribution of the mononuclear cells that make up the convoluted tubules (Figure 2a). As regards the TZ group (Figure 2b), a loss of the normal organization of the mononuclear cells was observed, with the presence of erythrocytes and cellular infiltrates. This suggests an alteration in the renal cells, leading to increased levels of urea and creatinine (Table 3). In the NG group (Figure 2c), no structural changes were observed in the convoluted tubules. In the TZ + NG group, the presence of erythrocytes and a preserved structure of the tubular cells were observed (Figure 2d).

The third section presents the analysis of histological sections of the intestine (Figure 3), in which the VEH group revealed both a muscular layer and a layer connecting the crypts, with no morphological abnormalities. In addition, the crypts and valleys exhibited a normal structure (Figure 2a). Regarding the TZ group (Figure 3b), a partial loss of tissue integrity was observed at the level of the crests, along with the presence of cellular infiltrates in the muscle tissue of the longitudinal layer, as well as thickening of this tissue. In the NG group (Figure 3c), no structural changes were observed in the crypts, valleys, or muscle layers. The TZ + NG group showed a preserved structure of the muscle layers and the layers connecting to the crests and valleys (Figure 3d).

## 4. Discussion

The objective of the study was to evaluate the toxicity induced by TZ and its potential attenuation with NG treatment in rats. Chronic administration of TZ resulted in motor alterations, which were attenuated by NG, which may be associated with the hepato- and nephroprotective effects exerted by NG because of its antioxidant effects. In the kidney and liver function tests, the groups treated with TZ presented values above the biological reference intervals, whereas the combination of TZ + NG maintained values within these intervals, indicating that NG prevents the toxicity of TZ. Finally, in the histological analysis, damage to liver, kidney, and intestinal tissues was observed in the TZ groups, whereas treatment with NG mitigated the cellular damage caused by TZ.

### 4.1. Behavioral Analysis

The motor activity test allowed the identification of a state of hypoactivity in the rats, which was evidenced by the reduction in the number of crossings. Thus, our results coincide with previous studies where male Wistar rats, which were administered erythrosine or tartrazine, showed a significant reduction in locomotion [50]. This hypoactivity could be related to neurological damage as reported in behavioral studies when toxic substances were administered [51], which also occurs with administration of TZ [4,19]. This effect has been related to cellular damage in specific brain structures like prefrontal cortex, cerebellum, corpus striatum, and hippocampus due to an increase in the oxidative stress, pro-inflammatory interleukins, and lipid peroxidation [4].

A significant reduction in the number of rearing behaviors, but not in the time spent rearing behavior, was observed. In contrast, a study conducted in newborn mice administered 2.5 mg/kg and 5 mg/kg TZ revealed a decrease in both the number and time of rearing behaviors [22]. This discrepancy in our results may be related to differences in the age of experimental subjects in which TZ was administered. This effect of TZ in the number of rearing behaviors could be the result of cellar damage in the above indicated brain structures, constituting a part of the neurological damage suggested here.

On the other hand, there was a significant reduction in the number of grooming behaviors in the TZ-treated rats in the present study. Grooming behavior can be used as a measure of the emotional state of the animals. When grooming behavior is reduced versus control groups, it is considered as an indicator of amotivation; contrarily, when grooming behavior is similar to control groups, it indicates a normal emotional state. Thus, the results of the present study suggest an emotional maladjustment that could be associated with neurological impairment. In this way, the exposure of animals to TZ caused alterations in the striatum, resulting in a reduction of motivation in the rats [52], which partially explains the reduction of the number of grooming behaviors in the present study. Similarly, rats treated with erythrosine and TZ (2 mg/kg, 6 mg/kg, and 10 mg/kg, b.w., p.o, 50:50) exhibited irregular behavior associated with alterations in the striatum in the experimental subjects [50].

In addition, a reduction in the latency to fall was observed in animals treated with TZ, which may be related to motor impairment as observed in the locomotor activity test. In this way, our results are like those of a study conducted in male rats that were administered homocysteine and other toxics which induced a cascade of excitotoxicity and ROS production [53,54], resulting in an impairment of motor coordination.

It is important to highlight that NG partially protected the rats against motor alterations induced by TZ. The treatment with NG attenuated the negative effect of TZ on crossing (locomotion) and latency to fall (motor coordination), which suggest that NG protects against motors impairment but not on emotional and exploration behavior. These effects of NG could be associated with their antioxidant and anti-inflammatory activity [55,56,57], which reduces the cellular damage in the brain structures that participate in the integration and expression of behavior, reducing the negative effects of TZ.

On the other hand, the biochemical and histological alterations in liver and renal function may alter the metabolism and elimination of unnecessary substances for the body. The changes in GGT, AST, ALT, urea, and creatinine can cross the blood–brain barrier and induces oxidative stress, pro-inflammatory, and lipid peroxidation [36,58]; which may produce cellular damage in the brain structures that participate in the integration and expression of behavior. Thus, consideration of the protective effects of NG on liver and renal function may reduce the damage of brain structures and partially attenuate the motor and coordination impairment. However, specific studies are required to test or refute this hypothesis.

### 4.2. Effects of Tartrazine and Naringenin on Liver Function

The assessment of liver function in experimental models is essential for determining the extent of damage induced by hepatotoxic agents and for evaluating the protective effect of compounds in preclinical studies. To this end, serum biochemical parameters are used, supplemented by histological analysis of liver tissue [59,60]. The results proposed by Khaled et al. (2023) are like those which we report [61], where Wistar rats were administered Taxol intraperitoneally before receiving oral NG treatment every two days for 42 days at a dosage of 10 mg/kg. This resulted in a significant improvement due to the damage produced by Taxol, which was reflected in the decrease in the values of the analytes that were measured by liver function tests. On the other hand, Kahramanoğullari et al. (2024) [62] administered NG and mercury chloride to 2-month-old male Wistar rats and reported a decrease in liver function This is consistent with the results found in our study. In this regard, the elevation of the aforementioned parameters, accompanied by histopathological lesions in vital organs such as the liver, kidneys, and intestines, is similar to that which we reported in our results [7,8,30,46,63,64,65,66,67]. Experimental models have confirmed naringenin’s hepatoprotective effects against various toxins. For example, in cadmium-induced toxicity, repeated administration of naringenin reduced serum markers of liver damage and improved the antioxidant status of the tissue [68]. In models of lead exposure, treatment with naringenin reduced hepatic biochemical abnormalities and oxidative stress [69]. Similarly, in ethanol-induced liver damage, naringenin normalized ALT and AST levels, improved hepatic antioxidant status, and reduced the inflammatory response [70]. Naringenin also demonstrated antifibrotic effects in a model of induced liver fibrosis, where it slowed fibrotic progression and modulated relevant profibrogenic pathways [71]. Various preclinical studies evaluating the hepatoprotective effects of naringenin primarily use the oral route, either via intragastric administration or mixed with vehicles such as DMSO diluted in saline, corn oil, or carboxymethylcellulose. Reported doses range from 25 to 200 mg/kg/day in acute, subacute, and subchronic regimens, depending on the hepatotoxic model used [32,68,70,71,72]. In functional terms, naringenin administration produces a consistent reduction in ALT, AST, and MDA, accompanied by an increase in GSH, SOD, CAT, and GPx, demonstrating restoration of the hepatic antioxidant system. Furthermore, histological analyses confirm preservation of the hepatic parenchyma, with less necrosis, reduced inflammatory infiltrate, and maintenance of hepatocellular architecture compared to the intoxicated groups [32,60,70,71,72]. In general, experimental models agree that naringenin exerts hepatoprotection mediated by antioxidant, anti-inflammatory, and anti-apoptotic mechanisms.

In this sense, naringenin has hepatoprotective potential, which is attributed to its ability to neutralize ROS through increased expression and activation of antioxidant enzymes, as well as to modify the enzymatic activity of gamma-glutamylcysteine synthetase (GGT), which acts in the GSH synthesis pathway. Wu et al. (1998) reported that naringenin possesses anti-inflammatory properties [73]. Taking medications and, as a result, the production of metabolic toxins in the body can facilitate the generation and release of ROS, cytokines, and other inflammatory factors. This activates Kupffer cells in the liver, leading to the destruction of hepatocytes. Hepatic stellate cells (HSCs) play a role in the formation of basement membranes, as they produce extracellular matrix components such as type IV collagen and laminin; Furthermore, they undergo phenotypic changes, proliferation, and excessive production of the extracellular matrix when exposed to factors released by stimulated Kupffer cells, resulting in liver fibrosis. Naringenin demonstrated hepatoprotective and antifibrotic effects against dimethylnitrosamine (DMN)-mediated liver damage in rats, while oral administration of naringenin resulted in decreased levels of GOT, GPT, alkaline phosphatase, and bilirubin [36]. Naringenin was reported to reduce the activation of Kupffer cells, thereby counteracting inflammatory changes in the liver caused by a high-cholesterol diet. Furthermore, naringenin regulates levels of inflammation and necrosis by degrading the extracellular matrix [36].

In addition to this, Sahu et al. (2019) induced hepatotoxicity with antituberculosis drugs in patients; by administering naringenin at three different doses (10, 20, and 40 mg/kg); they were able to restore serological parameters to control levels in a dose-dependent manner [74]. The 20 and 40 mg/kg doses of naringenin were significantly effective in restoring TGP, TGO, and ALP to control levels. Arefin et al. described cases of hepatotoxicity and nephrotoxicity at doses of 200–400 mg/kg, including significant increases in bilirubin, creatinine, and body weight [75].

ROS production may be the primary cause of hepatotoxicity, which can lead to various liver diseases. ROS induces cell membrane peroxidation and causes cellular damage, which manifests as the release of enzymes such as TGO and TGP. Free radicals are also believed to be involved in the activation of caspases-3, -8, and -9, leading to the induction of apoptosis during liver failure [36]. ROS induces cell membrane peroxidation and causes cellular damage that ultimately leads to the leaching of enzymes such as TGO and TGP. For this and other reasons, the need arose to identify a substance that would have a beneficial effect or at least counteract the damage [76]. Based on the above, the hepatoprotective potential of naringenin is attributed to its ability to counteract the effects of ROS by increasing the expression and activation of antioxidant enzymes. Furthermore, it was observed that naringenin can modulate the enzymatic activity of gamma-glutamylcysteine synthetase (GGT), which is involved in glutathione (GSH) synthesis.

### 4.3. Effects of Tartrazine and Naringenin on Renal Function

The most commonly used tests for renal function in experimental models include serum biochemical parameters and histological analysis of renal tissue. Biochemically, the most widely used biomarkers are serum urea and creatinine, whose levels rise when there is a decrease in the glomerular filtration rate [77,78].

The results reported by Khayyat et al. (2017) are like ours [18]. They orally administered 7.5 mg/kg TZ for 4 weeks and reported elevated serum creatinine and urea levels in the group treated with TZ in comparison with controls [18]. On the other hand, Kahramanoğullari et al. (2024) administered NG and mercury chloride to 2-month-old male Wistar rats and reported decreases in plasma urea and creatinine levels, which coincided with the values obtained in the different parameters that make up the renal profile, since NG reverses the toxicity of TZ [62]. Our results are consistent with those reported by Ammar et al. (2018), who found a significant increase in serum creatinine and urea levels in rats following oral administration of CCl_4_ [79]. Furthermore, subchronic exposure to TZ has been linked to alterations in liver and kidney parameters, as evidenced by increases in alanine aminotransferase (ALT), aspartate aminotransferase (AST), urea, and total protein [12], as well as transaminases, lactate dehydrogenase (LDH), creatinine, and uric acid, along with decreases in total protein, albumin, and globulins [6,80,81]. Furthermore, naringenin has demonstrated nephroprotective effects in various preclinical models of renal toxicity induced by gentamicin, cisplatin, and other agents that induce oxidative stress [37,38,39,40,41,42,43]. Its activity has been linked to a reduction in lipid peroxidation, the modulation of proinflammatory cytokines, and the inhibition of apoptotic pathways. In models of cisplatin-induced nephrotoxicity, intraperitoneal administration of naringenin at 50 mg/kg/day for 10 days, using DMSO diluted in saline as a vehicle, decreased caspase-3 expression, inhibited NF-κB, and reduced tubular apoptosis [40]. Other studies have reported similar findings, including attenuation of the inflammatory infiltrate and preservation of glomerular and tubular morphology [38,42,43]. Overall, preclinical evidence shows that naringenin exerts nephroprotective effects when administered both orally and intraperitoneally, at doses ranging from 25 to 50 mg/kg/day and for 7 to 10 days, with consistent effects on renal function, oxidative balance, and the structural integrity of the tubular epithelium [37,38,39,40,41,42,43]. Nephrotoxicity is often attributed to inflammation of the renal tubules, glomeruli, and the surrounding cellular matrix due to oxidative stress and the overproduction of ROS [82]. In this regard, naringenin reduces the methotrexate-induced increase in caspase-3 expression levels in renal tissue by exerting an anti-apoptotic effect through the modulation of NF-κB, p53, Bcl-2, Bax, and caspase-3. Similarly, administration of naringenin increases Bcl-2 expression in kidney tissue by >24.7%, suggesting increased inhibition of the mitochondrial pathway of cell death.

Therefore, naringenin exhibited a protective effect, as it restored liver and kidney parameters to normal levels and demonstrated a predominant preservation of the histological architecture of the liver, kidney, and intestine. In this regard, the observed protective effect could be related to the antioxidant, anti-inflammatory, and anti-apoptotic properties described for naringenin. Zaidun et al. (2018) noted that this flavanone plays a role in modulating processes associated with oxidative stress [31]. For their part, Wang et al. (2014) [32], Alam and Najum us Saqib (2020) [68], and Ali et al. (2022) [83] described a decrease in malondialdehyde, restoration of antioxidant defenses such as SOD, CAT, and GSH, and inhibition of inflammatory mediators such as TNF-α, IL-6, NF-κB, iNOS, and COX-2 in models of toxin-induced liver damage.

### 4.4. Histological Analysis

Khayyat et al. (2017) [18] conducted a study in Wistar rats which were orally administered 7.5 mg/kg TZ for 1 month and reported morphological changes in hepatic cytoarchitecture due to necrotic processes in hepatocytes, in addition to congestion of the hepatic central artery and leukocytic infiltration. In addition, Himri et al. (2011) [84] reported that the oral administration of TZ to Wistar rats at doses of 5, 7.5, and 10 mg/kg for 3 months caused blood vessel congestion, cellular infiltrates, and hepatocyte apoptosis. Therefore, our results are similar to the damage caused by TZ, which indicates a hepatotoxic effect, since a dilated central hepatic vein is observed in the presence of infiltrating cells. In addition, a study by Himri et al. (2011) [84] revealed that at a higher dose, the damage increased, resulting in the dilation and degeneration of glomerular tubules and capillaries, leading to renal dysfunction. Compared with our results for renal tissue in the TZ group, we observed a loss of density of cellular and hyperchromic nuclei that make up the convoluted tubules in the presence of infiltrating cells as well as a marked presence of erythrocytes.

On the other hand, Yan et al. (2023) [85] intraperitoneally administered an agonist of the aryl hydrocarbon receptor (CH223191) (10 mg/kg) and subsequently administered NG (50 mg/kg) intraperitoneally. The results showed that NG decreased the involvement of the intestinal lesion caused by CH223191 (rupture of the intestinal villi and mucosal lesions, with a clear increase in inflammation). In comparison with our results, we observed a loss of tissue integrity at the level of the crests, together with the presence of cellular infiltrates in the muscle tissue of the longitudinal layer, as well as thickening of this tissue and the presence of cellular infiltrates in the TZ group. In contrast, in the NG groups, no structural changes were observed in the crypts, the valleys, or the muscle layers.

In this study we did not evaluate markers of oxidative stress, inflammation, or antioxidant defense. Naringenin is a flavonoid that, in particular, may exhibit pro-oxidant activity under certain conditions. This activity may be beneficial by inducing mild oxidative stress, which enhances antioxidant defense mechanisms, as has been reported for naringenin, which, thanks to its pro-oxidant potential, can act as an anticancer molecule by inducing apoptosis in cancer cells [86]. In this regard, naringenin exerts a modulatory effect on pro-inflammatory and anti-inflammatory cytokines [55,56,57]. Furthermore, several documented studies have demonstrated naringenin’s significant potential to induce apoptosis by downregulating Akt and activating caspase-3 in both in vivo and in vitro studies [87,88,89]. However, further research is still needed to validate the use of naringenin as a potential candidate for improving renal and hepatic function, based on studies of its possible mechanisms [76].

### 4.5. Limitations and Strengths

The main limitations of this study include: the following: (1) Levels of Nrf2 and NF-κB, as well as the gene expression of antioxidant enzymes, were not determined. Therefore, a further study incorporating these molecular analyses is necessary to gain a deeper understanding and confirm the exact mechanism or mechanisms by which naringenin exerts its protective effect; (2) A histological analysis of brain tissue—particularly the striatum—was not performed and we will take this into account for future studies; (3) the exclusive use of male rats; (4) uncertainty regarding the translational relevance of the high dose of tartrazine; (5) the lack of direct measurements of oxidative stress; (6) the absence of pharmacokinetic measurements.

Although various preclinical studies have demonstrated the protective effect of naringenin on the liver and kidneys, there are still no clinical studies that have evaluated this effect; therefore, we propose conducting a future evaluation of a potential herbal medicine or pharmaceutical formulation based on this flavanone, aimed at reducing liver and kidney damage associated with the administration of drugs or food additives with potential hepatotoxic and/or nephrotoxic effects. Furthermore, given the risk of cumulative exposure to tartrazine, individual variability, and the lack of clarity regarding chronic toxicity thresholds, it is appropriate and necessary to reassess the safety of this food additive, and therefore priority should be given to replace tartrazine with safer natural alternatives in order to protect public health, especially among vulnerable populations such as children.

Despite some limitations of this study, it has several strengths: First, this study presents three levels of analysis related to tartrazine toxicity and the beneficial effects of naringenin (i.e., behavioral, biochemical, and histological analyses in the same animal); second, it is possible to discuss the associations between the behavioral, biochemical, and histological results of different organs to explain both the toxic and beneficial effects of naringenin in the same animal. Thus, these data help elucidate the partial mechanism of action of tartrazine and the beneficial effects of naringenin on tartrazine-induced toxicity. These results open new lines of investigation focused on identifying specific toxic effects of tartrazine, pharmacological interactions, and the beneficial effects of naringenin to ensure the appropriate regulation of its use in humans.

## 5. Conclusions

The present study revealed that naringenin produced a protective effect against tartrazine treatment in adult male rats, as it restored liver and kidney biochemical parameters to normal levels and demonstrated predominant preservation of the histological architecture of the liver, kidney, and intestine, which was associated with better behavioral ejection of tartrazine-intoxicated rats. This protective effect may be related to the antioxidant, anti-inflammatory, and anti-apoptotic properties described for naringenin. However, further research through mechanistic studies is needed to validate naringenin as a potential candidate for improving renal and hepatic function. Although the present study did not quantify markers of oxidative stress, inflammation, or apoptosis, this hypothesis should be considered a likely pathophysiological explanation, which is supported by the literature.

## Figures and Tables

**Figure 1:**
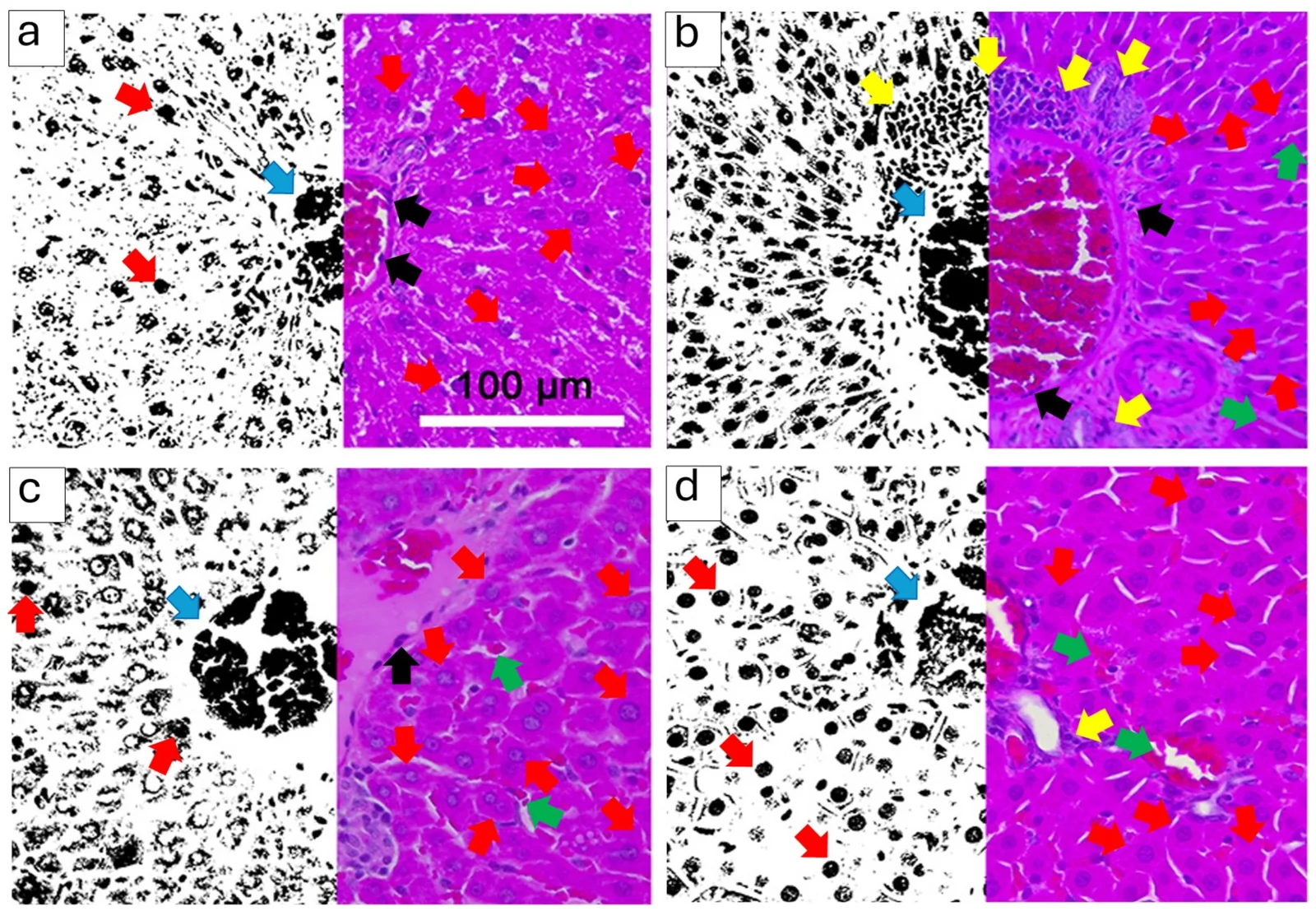
H&E-stained liver micrograph. (**a**) VEH group: Preserved hepatic architecture is observed, with the central hepatic vein showing no dilation (blue arrow), hepatocytes (red arrows) and normal sinusoids (black arrows). (**b**) TZ group: Collapse of the central hepatic vein (blue arrow). Marked infiltration of inflammatory cells (yellow arrows), impaired integrity of the sinusoids (black arrows), hepatocytes with hypochromic nuclei (red arrows) are observed and erythrocytes are present (green arrows). (**c**) NG group: The central hepatic vein is observed with normal distribution (blue arrow). Hepatocytes retain their cellular integrity (red arrows), the sinusoids are normal (black arrows), and erythrocytes are present (green arrows). (**d**) TZ + NG group: Slight dilation of the central hepatic vein (blue arrow); hepatocytes with no alterations in cellular organization (red arrows) are observed, with the presence of erythrocytes (green arrows) and infiltrating cells (yellow arrow).

**Figure 2:**
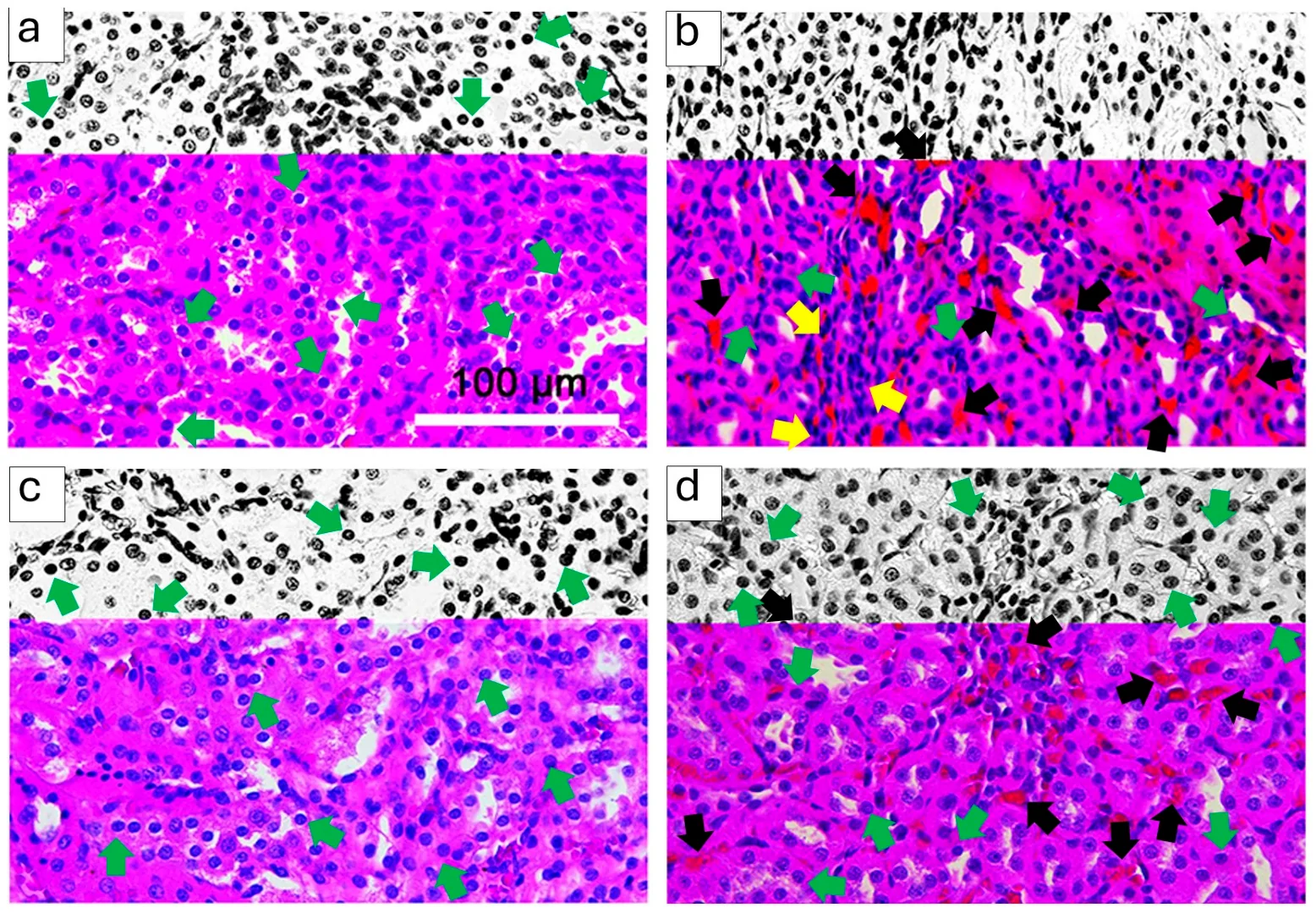
H&E-stained kidney micrograp. (**a**) VEH group: An intact distribution of mononuclear cell nuclei (green arrows) is observed in the convoluted tubules. (**b) TZ group**: Alteration and disorganization of the nuclei comprising the convoluted tubules (green arrows) is observed, with infiltration of inflammatory cells (yellow arrows); a fragmented structure with a marked presence of erythrocytes (black arrows) is evident. (**c**) NG group: The tubular architecture is intact and the nuclear distribution is preserved (green arrows), with no evident inflammatory infiltrate. (**d**) TZ + NG group: The structure of the renal cells is preserved (green arrows) with the presence of erythrocytes (black arrows).

**Figure 3:**
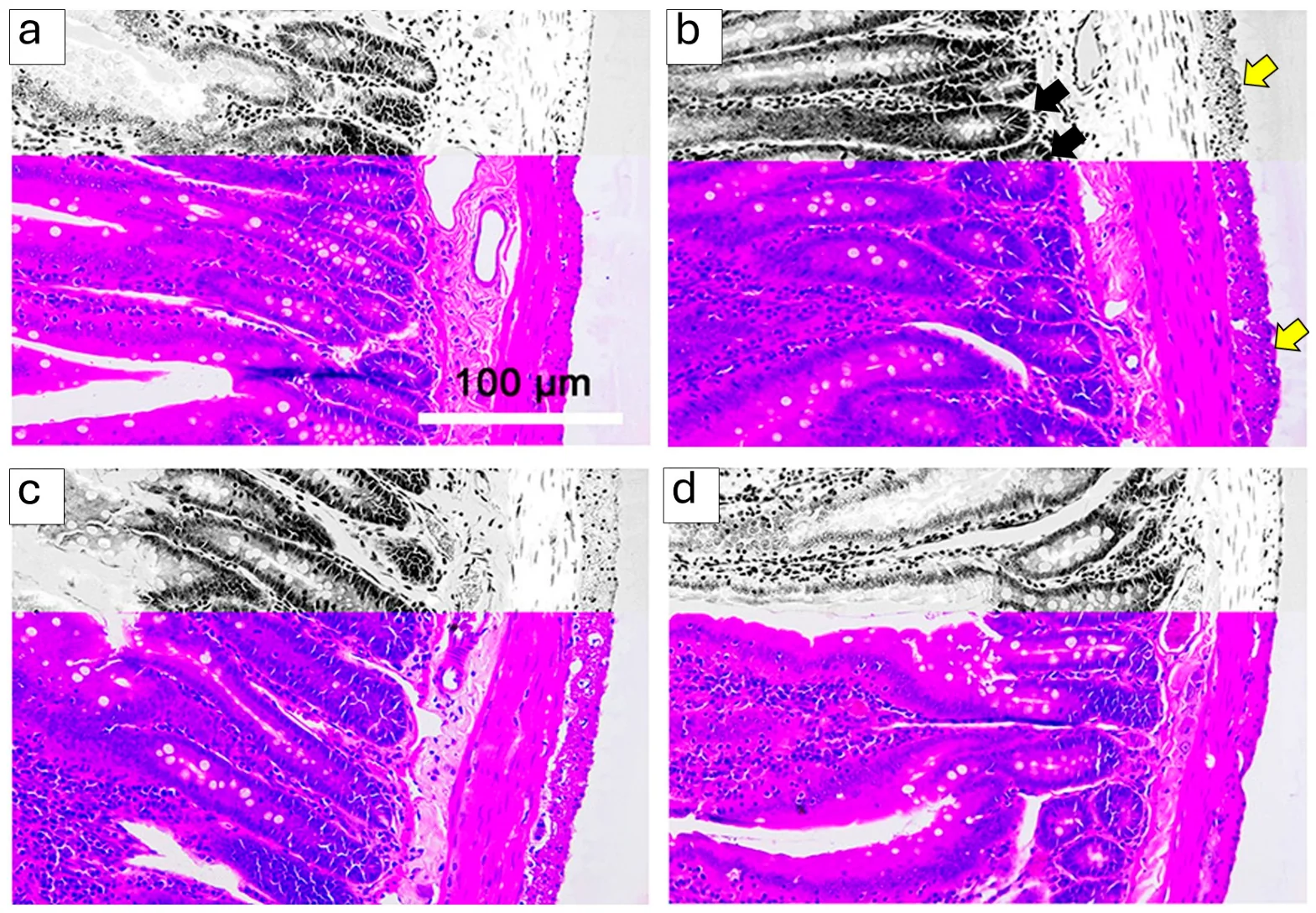
H&E-stained **intestine** micrograph: (**a**) **VEH group:** Intestinal structure preserved; the muscular layer and the layer connecting the crypts show no structural alterations. The crypts and valleys retain their structure with no infiltrates present. (**b**) **TZ group**: Intestinal structure showing loss of integrity of the crypts and valleys (black arrows), with cellular infiltrates present in the muscle tissue (yellow arrows). (**c**) **NG group**: Intestinal structure preserved; the crypts and valleys retain their structure with no infiltrates present. (**d**) **NG + TZ group**: Intestinal structure preserved; the crypts and valleys retain their structure with no infiltrates present.

**Table 1:** Variables evaluated in the behavioral test.

Locomotor Activity Test				
Group/Variable	Vehicle (VEH)	Tartrazine (TZ)	Naringenin (NG)	TZ + NG
Crossing (n)	43.42 ± 3.21	23.42 ± 2.20 *	34.00 ± 4.18 ^NS^	33.57 ± 1.98 ^NS^
Rearing behavior (n)	23.00 ± 1.91	9.28 ± 0.86 *	13.85 ± 3.35 *	12.71 ± 1.16 *
Rearing behavior (s)	96.71 ± 18.59	123.14 ± 18.11 ^NS^	79.14 ± 10.71 ^NS^	86.00 ± 7.71 ^NS^
Grooming behavior (n)	8.85 ± 1.79	2.57 ± 0.36 ^+^	7.00 ± 0.65	6.42 ± 0.68
Grooming behavior (s)	47.85 ± 12.51	49.71 ± 14.70 ^NS^	68.28 ± 17.04 ^NS^	75.00 ± 21.47 ^NS^
**Rota-Rod Test**				
**Group/Variable**	**Vehicle (VEH)**	**Tartrazine (TZ)**	**Naringenin (NG)**	**TZ + NG**
Latency to fall (s)	218.24 ± 24.61	44.14 ± 10.06 ^&^	179.57 ± 46.24	129.72 ± 30.17 *

* *p* < 0.05 vs. VEH group, ^+^ *p* < 0.05 vs. VEH, NG, and TZ + NG groups, ^&^ *p* < 0.05 vs. VEH and NG groups, NS, not significant. One-way ANOVA, post hoc Student–Newman–Keuls or Kruskal–Wallys tests, post hoc test Dunn. n, number; s, time in seconds; VEH, vehicle group; TZ, tartrazine group; NG, naringenin group; TZ + NG, tartrazine plus naringenin group.

**Table 2:** Liver function tests.

Parameters	Vehicle (VEH)	Tartrazine (TZ)	Naringenin (NG)	TZ + NG	Biological References Intervals
Gamma glutamyl transferase (GGT)	12.73 ± 1.37	33.39 ± 2.79 *	12.60 ± 1.14	14.76 ± 0.99	8.8–24 UI/L
Total proteins (TP)	6.95 ± 0.23	4.96 ± 0.12 *	5.85 ± 0.12	6.01 ± 0.18	5.60–7.60 g/dL
Alkaline phosphatase (ALP)	106.40 ± 20.66	347.80 ± 8.01 *	150.40 ± 33.48	183.70 ± 20.62	36–312 UI/L
Aspartate aminotransferase (AST)	86.63 ± 10.50	249.80 ± 26.97 *	91.99 ± 14.01	69.31 ± 2.01	63–157 UI/L
Alanine aminotransferase (ALT)	40.15 ± 4.76	84.78 ± 12.78 *	34.86 ± 6.06	33.54 ± 3.82	19–53 UI/L
Total bilirubin	0.12 ± 0.01	0.72 ± 0.07 *	0.11 ± 0.02	0.12 ± 0.01	0.04–0.20 mg/dL
Direct bilirubin	0.04 ± 0.01	0.23 ± 0.06 *	0.05 ± 0.01	0.04 ± 0.01	0.03–0.06 mg/dL
Indirect bilirubin	0.06 ± 0.01	0.44 ± 0.08 *	0.05 ± 0.01	0.06 ± 0.01	0–0.10 mg/dL

* *p* < 0.05 vs. VEH, NG, TZ + NG, one-way ANOVA and post hoc Student Newman tests. VEH, vehicle group; TZ, tartrazine group; NG, naringenin group; TZ + NG, tartrazine plus naringenin group.

**Table 3:** Kidney function tests.

Parameters	Vehicle (VEH)	Tartrazine (TZ)	Naringenin (NG)	TZ + NG	Biological References Intervals
Urea	15.73 ± 0.95	36.57 ± 3.79 *	15.24 ± 1.43	16.17 ± 1.10	10.70–20 mmol/L
Creatinine	14.70 ± 1.09	34.74 ±1.11 *	19.00 ±1.64	15.63 ±1.34	11–28 µmol/L

* *p* < 0.05, compared with the vehicle group (one-way ANOVA for independent groups followed by the Student-Newman-Keuls post hoc test). Source: Rivadeneyra et al., 2018 [49].

## Data Availability

The data will be made available by the corresponding authors upon request due to the project privacy requirement before closing the project.

## Abbreviations

The following abbreviations are used in this manuscript:

3R	Russell’s 3 Rs
ADI	Acceptable daily intake
Akt	Protein Kinase B
ALP	Alkaline phosphatase
ALT	Alanine aminotransferase
ANOVA	Analysis of variance
AST	Aspartate aminotransferase
Bax	Promotes apoptosis by competing with Bcl-2
Bcl-2	Anti-apoptotic factor
CAT	Catalase
CCl_4_	Carbon tetrachloride
COX-2	Cyclooxygenase-2
DMN	Dimethyl nitrosamine
DMSO	Dimethyl sulfoxide
DNA	Deoxyribonucleic acid
EFSA	European Food Safety Authority
EU	European Unión
GGT	Gamma glutamyl transferase
GOT	Glutamic-oxaloacetic transaminase
GPT	Glutamate pyruvate transaminase
GPX	Glutathione peroxidase
GSH	Glutathione
HSCs	Hepatic stellate cells
IL-1α	Interleukin 1α
IL-1β	Interleukin-1β
IL-6	Interleukin 6
INOS	Inducible nitric oxide synthase
IP	Intraperitoneal
JECFA	Joint FAO/WHO Expert Committee on Food Additives
LDH	Lactate dehydrogenase
MDA	Malondialdehyde
NBF	Neutral buffered formalin
NF-kB	Nuclear factor-κB
NG	Naringenin
NG–TZ	Naringenin–Tartrazine
NOM	Official Mexican standard
Nrf2	Nuclear factor erythroid 2-related factor 2
OS	Oxidative stress
P_53_	P53 tumor protein gene
PBS	Phosphate-buffered saline
ROS	Reactive oxygen species
SEM	Standard error of the mean
SOD	Superoxide dismutase
TNFα	Tumor Necrosis Factor Alpha
TP	Total protein
TZ	Tartrazine
VEH	Vehicle
WHO	World Health Organization
